# Gender-Specific Associations between Socioeconomic Status and Psychological Factors and Metabolic Syndrome in the Korean Population: Findings from the 2013 Korean National Health and Nutrition Examination Survey

**DOI:** 10.1155/2016/3973197

**Published:** 2016-12-05

**Authors:** Kyoung Im Cho, Bo Hyun Kim, Hyung Gon Je, Jae Sik Jang, Yong Hyun Park

**Affiliations:** ^1^Department of Internal Medicine, Kosin University School of Medicine, 34 Amnam-Dong, Seo-Ku, Busan 602-702, Republic of Korea; ^2^Department of Internal Medicine, Pusan National University Hospital and Biomedical Research Institute, 179 Gudeok-Ro, Seo-Gu, Busan 602-739, Republic of Korea; ^3^Department of Cardiovascular and Thoracic Surgery, Research Institute for Convergence of Biomedical Science and Technology, Pusan National University Yangsan Hospital, Yangsan 626-770, Republic of Korea; ^4^Department of Internal Medicine, Busan Paik Hospital, University of Inje College of Medicine, Busan 614-735, Republic of Korea; ^5^Department of Internal Medicine, Pusan National University Yangsan Hospital, Yangsan 626-770, Republic of Korea

## Abstract

We aimed to assess the gender-specific associations between psychological factors and socioeconomic status (SES) and metabolic syndrome (MetS) in Korean adults. We examined 4,689 Korean adults aged 20–79 years who participated in the 2013 Korean National Health Examination and Nutrition Survey. With regard to SES, occupation status (none, manual, and nonmanual), marital status (single, married, divorced, and widowed), and psychological factors (detection of stress, depressive symptoms, and suicidal thoughts) were determined via questionnaires. Compared with married men, single and divorced men exhibited ORs (95% confidence interval [CIs]) for MetS of 0.45 (0.31–0.65) and 1.61 (1.02–2.55), respectively, after adjusting for covariates. However, this association was not significant in women. Compared with those in the lowest household income group and least educated group in women, the ORs for MetS in the highest income group and the most educated group were 0.63 (CI 0.46–0.86) and 0.46 (CI 0.32–0.67), respectively. Suicidal thoughts in men (OR 1.64, CI 1.03–2.61) and perceived stress in women (OR 1.26, CI 1.01–1.59) were associated with MetS. In this study, MetS has gender-specific associations with lower SES and psychological factors. Thus, gender-specific public health interventions based on SES and psychological factors are needed to prevent and treat MetS and reduce additional cardiovascular disease risk.

## 1. Introduction

Metabolic syndrome (MetS) is a cluster of several cardiometabolic risk factors including obesity with insulin resistance, impaired glucose tolerance, dyslipidemia, and high blood pressure. The prevalence of MetS is increasing worldwide and is now considered a serious health problem globally [1]. With improvements in the standard of living and lifestyle, the incidence of MetS is also markedly increasing. In Korea, rapid socioeconomic growth has resulted in profound lifestyle changes and an increase in obesity, which have both led to an increase in the prevalence of MetS from 24.9% in 1998 to 31.3% in 2007 [2, 3].

Patients with MetS are more susceptible to type 2 diabetes mellitus, cardiovascular disease (CVD), and some cancers, which are the leading causes of death in developed countries [4–6]. In addition, MetS does not only place a burden on the individual but also affect the economic and social aspects of society. Genetic, metabolic, and various environmental and social factors have been considered as risk factor for MetS. Hence, the identification of socioeconomic characteristics associated with MetS is essential for successful primary prevention [7, 8]. Moreover, social factors such as education level, occupation, household income, and marital status may influence MetS [9]. In fact, several researchers have reported on the association between socioeconomic status (SES) and the risk of MetS [10–19]. Cases with a lower SES more frequently present with MetS [10, 11]. In addition, depression and MetS have been found to be significantly correlated [12]. Furthermore, gender also reportedly influences the association of SES with MetS [13, 14]. However, their associations with MetS and its components in a large population of men and women remain unclear, due to conflicting results in different countries [11–17]. More importantly, only limited data are available for marital status as a risk factor of MetS, even though marital status may affect MetS differently in both genders [17, 18].

Although there is a reported association between SES and psychological factors and MetS in different countries, only a few studies have used national level data from the Korean population [19, 20]. Recently, Park et al. [19] reported that SES factors such as household income had a different influence between the sexes, wherein a positive association was noted for men and an inverse association was observed for women. Nevertheless, to the best of our knowledge, no comprehensive study has been conducted on the relationship of MetS with psychological factors and all SES variables, including marital status, occupation, household income, and education level, based on gender differences in the Korean population.

In the present study, we aimed to examine the gender-specific relationship of psychological factors and SES with MetS prevalence by using representative data for Korean men and women aged >20 years who participated in the Korean National Health Examination and Nutrition Survey (KNHANES) in 2013.

## 2. Materials and Methods

### 2.1. Study Population

This study was based on data obtained by the Sixth Korea National Health and Nutrition Examination Survey (KNHANES VI-1) performed in 2013, which is a nationwide, population-based, and cross-sectional representative survey to assess the health and nutritional status of Koreans, conducted by the Korea Centers for Disease Control and Prevention. The sampling units included households selected through a stratified, multistage, probability-sampling design, based on geographic area, sex, and age group by using household registries. The target population included all noninstitutionalized civilians aged >1 year in Korea. Of a total of 10,113 individuals who were sampled for the KNHANES VI-1A, a total of 8,018 individuals participated in the Health Behavior, Health Examination, or Nutrition surveys; the response rate was 79.3%. We used data from a Health Examination Survey, and informed consent was obtained during the interview. The survey was approved by the research ethical review board of the Korean Centers for Disease Control and Prevention. Subjects aged ≥20 years were selected from the KNHANES VI-1 population (*n* = 6113). Of these 6113 subjects, individuals without laboratory data such as lipid and fasting plasma glucose (FPG) levels and those who had not fasted for at least 12 hours prior to blood sampling (*n* = 623) were excluded. Subjects with chronic conditions, including any type of cancer (*n* = 223), white blood cell count >10,000 cells/mL (*n* = 155), liver cirrhosis (*n* = 16), chronic hepatitis (*n* = 282), renal failure (*n* = 22), myocardial infarction or angina (*n* = 114), or stroke (*n* = 149), were also excluded. Moreover, subjects lacking sufficient demographic data, including age, sex, waist circumference, body mass index (BMI), physical activity, smoking and drinking history, income level, education level, and occupation, were also excluded (*n* = 141). All the subjects included in the study had blood sampling data. Finally, a total of 4689 subjects (2,024 men and 2,665 women) were included in the analysis.

### 2.2. Data Collection

The health examinations included medical history taking, physical examination, a questionnaire on health-related behavior, and anthropometric and biochemical measurements. Physical examinations were performed by trained medical staff following standardized procedures, and blood sampling was performed in all individuals aged ≥20 years. Participants were asked about health behaviors, including mental health, cigarette smoking, alcohol consumption, sleep pattern, physical activity, and dietary habits. Current smokers were defined as those who had smoked >5 packs of cigarettes during their lifetime and were smoking at the time of the survey. All other subjects were defined as nonsmokers. Alcohol consumption was assessed by questioning the subjects about their drinking behavior during the month before the interview. Heavy alcohol drinking was defined as ≥7 drinks at one time, more than twice a week, for men (≥5 drinks for women).

All subjects were questioned about whether they exercised with an intensity that left them sweating and with slight difficulty in breathing. Subjects who exercised regularly at a moderate intensity were asked about the frequency at which they exercised per week and the duration of each exercise session. Physical activity was classified according to the presence of regular exercise as moderate-intensity physical activity (requires a moderate amount of muscle fatigue and noticeably increased breathing rate) for a minimum of 30 minutes each day for 5 days/week or vigorous-intensity physical activity (requires a large amount of muscle fatigue and causes rapid breathing and a substantial increase in heart rate) for a minimum of 20 minutes each day for 3 days/week. In addition, subjects were questioned about the number of days they walked and the duration of walking each week; walking implementation was defined as an implemented rate of walking for a minimum of 30 minutes each day for 5 days/week. Dietary intake was collected via a 24-hour recall method. Before testing, all the subjects were instructed to maintain their usual dietary habits. If they were being treated for any disease, they were asked for data on the diagnosis and a list of medications being taken. The completed questionnaires were reviewed by trained staff and the records were entered into a database.

The subjects' weights and heights were measured while they wore light clothing and after they had removed their shoes. BMI was calculated by dividing the weight by the square of height (kg/m^2^). Blood pressure (BP) was measured on the right arm using a standard mercury sphygmomanometer (Baumanometer, USA), with the patient in the sitting position. Systolic and diastolic BP (SBP and DBP,  resp.) readings were recorded twice at 5-min intervals and were averaged for analysis.

Blood samples were collected from the antecubital vein of each participant after overnight fasting. The samples were processed, transported to the Central Testing Institute located in Seoul (Korea), and analyzed within 24 hours. The levels of FPG, triglycerides (TG), high-density lipoprotein (HDL) cholesterol, total cholesterol, and low-density lipoprotein (LDL) cholesterol were measured using a COBAS 8000 C702 Chemistry Analyzer (Roche, Germany). LDL cholesterol levels were directly measured by using a homogenous enzymatic colorimetric method with the reagent LDL-C (Roche, Germany) instead of with the Friedewald calculation. Participants provided written informed consent for the use of their blood samples in further analyses.

### 2.3. Survey of Socioeconomic Status

Subjects were asked to provide data on the socio-demographic factors including household income, marital status, occupation, and educational level. Household-equalized income (total household income/household size^0.5^) was categorized into quartiles by gender as lowest, medium-lowest, medium-highest, and highest [20]. Occupation status was classified as none, manual, and nonmanual according to the Registrar General Social Classification by using data from a self-reported questionnaire; the occupation status of housewives was categorized as none. Marital status was classified as single, married, divorced, and widowed. Education level was classified based on the number of school years: elementary or less (≤6 years), middle school (7–9 years), high school (10–12 years), or college and above (≥13 years). Completed questionnaires were reviewed by trained staff and the records were entered into a database.

### 2.4. Survey of Psychological Factors

Subjects were asked to provide information on their level of psychological stress (none, low, moderate, and extreme). Moreover, perceived stress was classified as none/low or moderate/extreme. A separate set of questionnaires assessed the workers for the presence of depressive symptoms and suicidal thoughts during the past year. To check for the presence of a depressed mood, respondents were asked, “During the past year, have you experienced any feelings of sadness or hopelessness that persisted for at least 2 weeks and disrupted your social life?” to which they responded either “Yes” or “No.” Respondents were also asked whether they experienced stress in their daily lives. One question related to suicidal thoughts was as follows, “During the past year, have you ever felt that you were willing to die?” to which they responded “Yes” or “No.”

### 2.5. Definition of Metabolic Syndrome

MetS was defined based on the Modified National Cholesterol Education Program Adult Treatment Panel III (NCEP-ATP III) guidelines, along with the waist circumference values for Koreans suggested by the Korean Society for the Study of Obesity [21, 22]. MetS was diagnosed in cases where the patient met ≥3 of the following 5 criteria: waist circumference ≥90 cm in males and ≥85 cm in females, TG ≥150 mg/dL, HDL cholesterol <40 mg/dL in males and <50 mg/dL in females, SBP ≥130 mmHg and/or DBP ≥85 mmHg, and FPG ≥100 mg/dL. Subjects who reported taking antihypertensive or antidiabetic medications were considered to have elevated BP or high FPG levels.

### 2.6. Statistical Analysis

In the KNHANES, samples were randomly selected by using a systematic sampling method. Statistical estimates were weighted to represent the total population of Korea. Adjusted weights for sex, age, and region were applied to data in order to obtain a representative sample of the Korean population. Complex sample analysis was performed based on an analysis plan file wherein specific weights, stratification variables, and primary sampling units were designated. Demographic, biochemical characteristics, and psychological factors of the study population were compared according to sex by using an independent, two-sample *t*-test for continuous variables, and the chi-square test for categorical variables. The MetS variables in the study population were compared according to SES by using the one-way analysis of variance (ANOVA) test for continuous variables. The odds ratios (ORs) and 95% confidence intervals (95% CIs) for MetS and psychological factors and SES were calculated by using multivariate logistic regression analysis after adjusting for other covariates. The collected data were analyzed by using SPSS Statistics version 18.0 (SPSS Inc., Chicago, IL, USA). All statistical tests were two-sided and a *p*-value of <0.05 represented statistical significance.

## 3. Results

### 3.1. Basic Characteristics of the Study Population

Table 1 shows the characteristics of the 2024 men and 2665 women enrolled; the mean age was 48.9 ± 15.8 years for the men and 49.0 ± 15.6 years for the women. Considering the modified ATP III criteria, the overall prevalence of MetS was determined to be 27.5%. In particular, the prevalence of MetS was 30.9% (626/2,024) for men and 24.8% (662/2,665) for women; thus, MetS was significantly more prevalent in men (*p* < 0.001).

### 3.2. Prevalence of Metabolic Syndrome according to the Socioeconomic Status

The mean age, BMI, waist circumference, SBP, DBP, FPG level, and TG level were significantly higher in men and women with MetS, whereas the HDL cholesterol level was lower in men and women with MetS, compared with those in men and women without MetS. The proportion of individuals who were heavy alcohol drinkers was significantly higher in men with MetS (32.1% versus 38.3%; *p* = 0.008) and significantly lower in women with MetS (7.9% versus 4.4%; *p* < 0.001). As expected, the MetS prevalence tended to decrease when physical activity, including vigorous-intensity exercise, moderate-intensity exercise, and walking implementation, increased in both sexes.

Significant differences in the association among marital status, occupational status, education, and MetS were observed between men and women. With regard to marital status, the MetS prevalence was higher in married, divorced, or widowed men but was higher only in widowed women. Both household income and education level were inversely associated with MetS prevalence in both sexes. However, the inverse association was more prominent in women. With regard to occupation, the MetS prevalence was significantly higher in unemployed women compared with that in employed women, whereas there was no significant association between MetS and occupation in men (Table 1).

### 3.3. Prevalence of Metabolic Syndrome according to Psychological Factors

Depressive symptoms and suicidal thoughts were associated with MetS in both sexes. However, perceived stress was associated with MetS only in women and not in men (Table 1).

### 3.4. Metabolic Syndrome Based on Socioeconomic Status and Psychological Factors

Table 2 presents the crude ORs (95% CI) of the prevalence of MetS with different SES variables in men and women. In general, all the SES variables were significantly associated with MetS in both sexes, except for occupation in men. Moreover, all the psychological factors were significantly associated with MetS in both sexes, except for perceived stress in men.

Table 3 shows the ORs of the prevalence of MetS with different SES variables in men and women after adjusting for age, smoking status, alcohol drinking status, and exercise status.

After adjusting for these important confounding variables, a significant relationship showing a high prevalence of MetS in divorced men was observed (OR, 1.61; 95% CI, 1.02–2.55; *p* = 0.042). However, there was no significant correlation between marital status and MetS in women. Moreover, occupation was not significantly associated with MetS in both sexes after adjusting for the covariables.

The MetS prevalence was lower in the highest income group (OR, 0.63; 95% CI, 0.46–0.86; *p* = 0.004), as compared to the lowest income group in women. A significant negative trend (*p* = 0.006) was observed in terms of income in women. However, no significant correlation between income group and MetS prevalence was noted in men. With regard to education level, the MetS prevalence was lower in the most well-educated subjects (OR, 0.46; 95% CI, 0.32–0.67; *p* < 0.001), compared to the least educated subjects, in women. In contrast, in men, no significant association was observed between SES variables and MetS prevalence, except for the marital status. Among the psychological factors, perceived stress was associated with MetS in women (OR, 1.26; 95% CI, 1.03–1.59; *p* = 0.047) and suicidal thoughts were associated with MetS in men (OR, 1.64; 95% CI, 1.03–2.61; *p* = 0.036).

## 4. Discussion

The principal findings of the present study include the gender differences in the relationship between SES and psychological factors and MetS prevalence. In particular, MetS was significantly more prevalent in men. Moreover, marital status was significantly related to MetS prevalence in men, but not in women. In contrast, a lower income and education status were related to MetS prevalence in women, but not in men. Occupation was not significantly associated with MetS in both sexes. Although depressive symptoms and suicidal thoughts were associated with MetS in both sexes, perceived stress was associated with MetS in women, but not in men. These findings suggest that gender-specific public health interventions that consider SES and psychological factors are needed to ensure appropriate MetS prevention and treatment and further reduce the risk of CVD.

MetS is one of the most common risk factors for CVD and mortality [1–4]. MetS is influenced by numerous factors including age, diet, eating habits, physical activity, gender, and genetic background. Thus, the identification and modification of these risk factors are very important for reducing the prevalence of MetS. Recently, a lower SES was found to be associated with CVD, type 2 diabetes, and MetS. Most prior studies have shown inverse relationships between SES and MetS. However, the association between SES and MetS showed conflicting results in different populations [10–19]. Santos et al. [13] reported that MetS was significantly more frequent in Portuguese women and that women in lower social classes (defined based on education status and occupational classification) more frequently presented MetS; no such association was found in men. Al-Daghri et al. [14] recently showed that MetS prevalence was more frequent in married, retired men from high-income households in Saudi Arabia. In contrast, higher education and income played an important role in decreasing the prevalence of MetS in women; no such effect was noted in men. In addition, Vernay et al. [15] stated that MetS prevalence decreased with an increasing education level in women, but not in men. Marital status and occupation were not found to be associated with MetS in both sexes in the French population. In the United States, Loucks et al. [16] also reported that a lower socioeconomic position was associated with MetS in women, an association that was weaker in men.

A comparison of the related risk factors of MetS across countries is difficult due to the differences in obesity prevalence, age groups, study population recruitment, and MetS definitions. Sex and racial/ethnic differences in the individual components of MetS have been reported previously, which suggests the possibility of varying contributions of the traditional components and a genetic component of MetS among these populations [23, 24]. Potential mechanisms linking SES to MetS include eating habits, physical activity, health behaviors, psychological distress, neighborhood characteristics, and access to health care [16]. Thus, we conducted a comprehensive analysis while adjusting for various covariates and considering the psychosocial factors. Although the findings of previous studies are not all consistent with our results, the common finding of a sex-specific association between MetS risk and SES was stronger in women than in men. Most of the previous studies have shown a stronger relationship between SES and MetS in women than in men [13–16]. Although the reasons for these gender differences between MetS and SES remain unclear, various factors have been proposed to explain them, including parity and consequent weight gain, obesity-related effects on social mobility, and greater concurrent psychosocial risks in women with lower SES than in men with lower SES [16]. In the present study, perceived stress was significantly associated with MetS in women. Future research that focuses on identifying the reasons for gender differences in the relationship between SES and MetS risk will help elucidate additional information regarding the underlying mechanisms.

An alteration in marital status may be associated with a change in health status. A recent study reported that marital transition might affect MetS risk differently according to sex and occupation [17]. Troxel et al. [18] reported that women in high-quality marriages were at lower risk of developing MetS. However, marital status was not independently associated with MetS in the Mediterranean population [11]. In the current study, marital status was significantly associated with MetS prevalence in men, but not in women. Divorced men tended to have greater rates of smoking, excessive alcohol drinking, physical inactivity, psychological distress, and poor eating habits. These inconsistent results may be due to differences in the study population and sociocultural factors. Moreover, most cross-sectional studies had not considered the duration of the change in marital status; hence, this parameter needs to be carefully assessed in a future prospective study. In addition, research efforts should be initiated to determine the socioeconomic risk factors of MetS while considering the differences in the sociocultural background and the appropriate target population in each country. These efforts could help prevent type 2 diabetes and CVD by raising the awareness of individualized risk factors in MetS patients and by aiding in establishing public health policies.

The present study has certain limitations. First, we were unable to determine a causal relationship between SES and psychological factors and MetS due to the cross-sectional study design. Second, we could not completely exclude the effects of information bias, as this study was based on a questionnaire survey. However, our study had multiple strengths, such as the large, representative sample of the Korean noninstitutional population; this promotes the applicability of the results to the entire Korean population. In addition, we comprehensively analyzed the association of SES and psychological factors with MetS after adjusting for almost all covariates. This analysis helped us to assess their association independent of the potential confounders.

In conclusion, SES and psychological factors have a considerable impact on the prevalence of MetS in Koreans, and their influences markedly differ between the sexes. Marital status was significantly related to the prevalence of MetS in men, but not in women. Moreover, the income and education status were related to the prevalence of MetS in women, but not in men. These findings suggest that gender-specific public health interventions that consider SES are needed for appropriate MetS prevention and treatment.

## Figures and Tables

**Table 1:** Characteristics of study population according to the presence of metabolic syndrome.

Variables	Men (*n* = 2024)			Women (*n* = 2665)		
	Without MS (*n* = 1398)	MS (*n* = 626)	*p*-value	Without MS (*n* = 2003)	MS (*n* = 662)	*p*-value
Age, years	47.0 ± 16.2	53.1 ± 14.1	<0.001	45.1 ± 14.5	61.0 ± 12.2	<0.001
BMI, kg/m^2^	23.3 ± 2.74	26.5 ± 3.17	<0.001	22.5 ± 2.97	26.2 ± 3.54	<0.001
Waist circumference, cm	81.1 ± 7.44	91.1 ± 7.89	<0.001	74.9 ± 8.06	86.6 ± 8.67	<0.001
SBP, mmHg	117.0 ± 14.0	127.7 ± 16.3	<0.001	111.4 ± 15.7	128.6 ± 16.9	<0.001
DBP, mmHg	76.0 ± 9.48	82.2 ± 12.4	<0.001	71.9 ± 9.42	77.0 ± 10.9	<0.001
FBG, mg/dL	95.9 ± 15.9	113.1 ± 26.6	<0.001	92.8 ± 14.7	111.9 ± 28.3	<0.001
Total cholesterol, mg/dL	186.0 ± 34.5	189.9 ± 39.2	0.033	187.8 ± 34.3	198.3 ± 39.3	<0.001
Triglycerides, mg/dL	121.7 ± 79.6	244.0 ± 190.5	<0.001	93.1 ± 49.8	185.7 ± 99.1	<0.001
HDL cholesterol, mg/dL	47.6 ± 9.68	39.2 ± 8.56	<0.001	53.2 ± 10.2	42.6 ± 7.55	<0.001
Current smoking (%)	552 (39.5%)	253 (40.4%)	0.364	98 (4.9%)	34 (5.1%)	0.426
Heavy alcohol consumption (%)	450 (32.1%)	241 (38.2%)	0.008	158 (7.9%)	29 (4.4%)	<0.001
Exercise						
Vigorous intensity	302 (21.6%)	96 (15.3%)	0.001	290 (14.5%)	55 (8.3%)	<0.001
Moderate intensity	126 (9.0%)	33 (5.3%)	0.002	102 (5.09%)	20 (3.02%)	0.017
Walking implementation	591 (42.3%)	233 (37.2%)	0.018	686 (34.2%)	204 (30.8%)	0.067
Activity limitations	89 (6.37%)	72 (11.5%)	<0.001	122 (6.09%)	95 (14.4%)	<0.001
Marital status			<0.001			<0.001
Single	317 (22.7%)	51 (8.1%)		316 (15.8%)	18 (2.7%)	
Married	1018 (72.8%)	521 (83.2%)		1430 (71.4%)	434 (65.6%)	
Divorced	42 (3.0%)	37 (5.9%)		98 (4.9%)	32 (4.8%)	
Widowed	21 (1.5%)	17 (2.7%)		159 (7.9%)	178 (26.9%)	
Household income			<0.001			<0.001
Lowest	205 (14.7%)	130 (20.8%)		275 (13.7%)	233 (35.2%)	
Medium lowest	357 (25.5%)	155 (24.8%)		541 (27.0%)	172 (26.0%)	
Medium highest	379 (27.1%)	178 (28.4%)		531 (26.5%)	151 (22.8%)	
Highest	456 (32.6%)	163 (26.0%)		656 (32.8%)	106 (16.0%)	
Education			<0.001			<0.001
Elementary or less (≤6 years)	184 (13.2%)	116 (18.5%)		360 (18.0%)	350 (52.9%)	
Middle school (7–9 years)	126 (9.01%)	83 (13.3%)		168 (8.39%)	87 (13.1%)	
High school (10–12 years)	535 (38.3%)	239 (38.2%)		729 (36.4%)	163 (24.6%)	
College and above (≥13 years)	553 (39.6%)	188 (30.0%)		746 (37.2%)	62 (9.37%)	
Occupation			0.120			<0.001
None	343 (24.5%)	163 (26.0%)		930 (46.4%)	400 (60.4%)	
Manual	581 (41.6%)	230 (36.7%)		781 (39.0%)	130 (19.6%)	
Nonmanual	474 (33.9%)	233 (37.2%)		292 (14.6%)	132 (19.9%)	
Psychological factors						
Perceived stress	281 (20.1%)	130 (20.8%)	0.386	458 (22.9%)	179 (27.0%)	0.015
Depressive symptoms	90 (6.4%)	57 (9.1%)	0.022	260 (13.0%)	119 (18.0%)	0.001
Suicidal thoughts	43 (3.1%)	36 (5.8%)	0.004	97 (4.8%)	51 (7.7%)	0.004

BMI, body mass index; SBP, systolic blood pressure; DBP, diastolic blood pressure; FBG, fasting blood glucose.

**Table 2:** Associations for the socioeconomic status, psychological factors, and metabolic syndrome.

Variables	Men			Women		
	OR^a^	95% CI	*p*	OR	95% CI	*p*
Age	1.03	1.03 to 1.03	<0.001	1.08	1.07 to 1.09	<0.001
Current smoking	1.04	0.86 to 1.26	0.706	1.06	0.71 to 1.58	0.790
Heavy alcohol consumption	0.85	0.69 to 1.05	0.127	0.46	0.38 to 0.56	<0.001
Marital status			<0.001			<0.001
Married	1			1		
Single	0.31	0.23 to 0.43	<0.001	0.19	0.12 to 0.31	<0.001
Widowed	1.58	0.83 to 3.02	0.168	3.71	2.92 to 4.71	<0.001
Divorced	1.72	1.09 to 2.70	0.020	1.08	0.71 to 1.63	0.733
Occupation			0.095			<0.001
None	1			1		
Nonmanual	0.96	0.75 to 1.22	0.739	0.39	0.31 to 0.48	<0.001
Manual	0.80	0.64 to 0.99	0.040	1.05	0.83 to 1.33	0.695
Economy			<0.001			<0.001
Lowest	1			1		
Medium lowest	0.68	0.51 to 0.91	0.009	0.37	0.29 to 0.47	<0.001
Medium highest	0.73	0.55 to 0.97	0.031	0.33	0.26 to 0.43	<0.001
Highest	0.54	0.41 to 0.72	<0.001	0.19	0.14 to 0.25	<0.001
Education			<0.001			<0.001
Elementary or less (≤6 years)	1			1		
Middle school (7–9 years)	1.05	0.73 to 1.50	0.797	0.53	0.40 to 0.72	<0.001
High school (10–12 years)	0.71	0.54 to 0.94	0.015	0.23	0.18 to 0.29	<0.001
College and above (≥13 years)	0.54	0.41 to 0.72	<0.001	0.09	0.06 to 0.12	<0.001
Exercise						
Severe intensity	0.66	0.51 to 0.85	0.001	0.54	0.40 to 0.73	<0.001
Moderate intensity	0.56	0.38 to 0.83	0.004	0.59	0.36 to 0.95	0.031
Walking	0.81	0.67 to 0.99	0.036	0.86	0.71 to 1.04	0.118
Activity limitation	1.89	1.36 to 2.61	<0.001	2.58	1.94 to 3.43	<0.001
Psychological factor						
Perceived stress	1.05	0.83 to 1.33	0.682	1.26	1.03 to 1.54	0.026
Depressive mood	1.45	1.03 to 2.05	0.034	1.48	1.16 to 1.87	0.001
Suicidal thoughts	1.92	1.22 to 3.02	0.005	1.64	1.16 to 2.33	0.006

^a^Crude odds ratio.

**Table 3:** Associations for the socioeconomic status and metabolic syndrome in men and women after adjustment for age, smoking status, alcohol drinking status, and exercise.

Variables	Men			Women		
	Adjusted OR	95% CI	*p*	Adjusted OR	95% CI	*p*
Marital status			<0.001			0.721
Married	1			1		
Single	0.45	0.31 to 0.65	<0.001	0.83	0.47 to 1.45	0.504
Widowed	1.34	0.69 to 2.60	0.168	0.96	0.72 to 1.29	0.796
Divorced	1.61	1.02 to 2.55	0.042	0.80	0.51 to 1.26	0.334
Occupation			0.430			0.780
None	1			1		
Nonmanual	1.19	0.91 to 1.56	0.198	0.91	0.71 to 1.18	0.483
Manual	1.13	0.87 to 1.45	0.357	0.97	0.75 to 1.26	0.804
Economy			0.364			0.006
Lowest	1			1		
Medium lowest	0.90	0.66 to 1.22	0.503	0.86	0.65 to 1.14	0.291
Medium highest	1.04	0.77 to 1.41	0.802	1.03	0.76 to 1.39	0.869
Highest	0.84	0.61 to 1.14	0.256	0.63	0.46 to 0.86	0.004
Education			0.304			0.001
Elementary or less (≤6 years)	1			1		
Middle school (7–9 years)	1.05	0.75 to 1.46	0.772	0.94	0.68 to 1.29	0.692
High school (10–12 years)	1.26	0.89 to 1.78	0.198	0.81	0.61 to 1.07	0.139
College and above (≥13 years)	1.21	0.96 to 1.52	0.102	0.46	0.32 to 0.67	<0.001
Psychological factor						
Perceived stress	1.25	0.98 to 1.59	0.076	1.26	1.003 to 1.59	0.047
Depressive mood	1.35	0.95 to 1.93	0.094	1.14	0.87 to 1.49	0.348
Suicidal thoughts	1.64	1.03 to 2.61	0.036	1.28	0.86 to 1.92	0.229

## References

[1] Park Y., Zhu S., Palaniappan L., Heshka S., Carnethon M. R., Heymsfield S. B. (2003). The metabolic syndrome: prevalence and associated risk factor findings in the US population from the Third National Health and Nutrition Examination Survey, 1988–1994. *Archives of Internal Medicine*.

[2] Lim S., Shin H., Song J. H. (2011). Increasing prevalence of metabolic syndrome in Korea: The Korean National Health and Nutrition Examination Survey for 1998–2007. *Diabetes Care*.

[3] Ha K. H., Kim D. J. (2015). Trends in the diabetes epidemic in Korea. *Endocrinology and Metabolism*.

[4] Wilson P. W., D’Agostino R. B., Parise H., Sullivan L., Meigs J. B. (2005). Metabolic syndrome as a precursor of cardiovascular disease and type 2 diabetes mellitus. *Circulation*.

[5] Lakka H., Laaksonen D. E., Lakka T. A. (2002). The metabolic syndrome and total and cardiovascular disease mortality in middle-aged men. *Journal of the American Medical Association*.

[6] Russo A., Autelitano M., Bisanti L. (2008). Metabolic syndrome and cancer risk. *European Journal of Cancer*.

[7] Lidfeldt J., Nyberg P., Nerbrand C., Samsioe G., Scherstén B., Agardh C. D. (2003). Socio-demographic and psychosocial factors are associated with features of the metabolic syndrome. The Women’s Health in the Lund Area (WHILA) study. *Diabetes, Obesity and Metabolism*.

[8] Minehira K., Tappy L. (2002). Dietary and lifestyle interventions in the management of the metabolic syndrome: present status and future perspective.. *European journal of clinical nutrition*.

[9] Booth S. L., Sallis J. F., Ritenbaugh C. (2001). Environmental and societal factors affect food choice and physical activity: Rationale, influences, and leverage points. *Nutrition Reviews*.

[10] Regidor E., Gutiérrez-Fisac J. L., Banegas J. R., López-García E., Rodríguez-Artalejo R. (2004). Obesity and socioeconomic position measured at three stages of the life course in the elderly. *European Journal of Clinical Nutrition*.

[11] Buckland G., Salas-Salvadó J., Roure E., Bulló M., Serra-Majem L. (2008). Sociodemographic risk factors associated with metabolic syndrome in a Mediterranean population. *Public Health Nutrition*.

[12] Pan A., Keum N., Okereke O. I. (2012). Bidirectional association between depression and metabolic syndrome: A systematic review and meta-analysis of epidemiological studies. *Diabetes Care*.

[13] Santos A. C., Ebrahim S., Barros H. (2008). Gender, socio-economic status and metabolic syndrome in middle-aged and old adults. *BMC Public Health*.

[14] Al-Daghri N. M., Alkharfy K. M., Al-Attas O. S. (2014). Gender-dependent associations between socioeconomic status and metabolic syndrome: a cross-sectional study in the adult Saudi population. *BMC Cardiovascular Disorders*.

[15] Vernay M., Salanave B., De Peretti C. (2013). Metabolic syndrome and socioeconomic status in France: The French Nutrition and Health Survey (ENNS, 2006-2007). *International Journal of Public Health*.

[16] Loucks E. B., Rehkopf D. H., Thurston R. C., Kawachi I. (2007). Socioeconomic disparities in metabolic syndrome differ by gender: evidence from NHANES III. *Annals of Epidemiology*.

[17] Hosseinpour-Niazi S., Mirmiran P., Hosseinpanah F., Fallah-ghohroudy A., Azizi F. (2014). Association of marital status and marital transition with metabolic syndrome: Tehran lipid and glucose study. *International Journal of Endocrinology and Metabolism*.

[18] Troxel W. M., Matthews K. A., Gallo L. C., Kuller L. H. (2005). Marital quality and occurrence of the metabolic syndrome in women. *Archives of Internal Medicine*.

[19] Park S., Kang H., Nam C., Park B., Linton J. A., Lee Y. (2012). Sex differences in the relationship between socioeconomic status and metabolic syndrome: The Korean National Health and Nutrition Examination Survey. *Diabetes Research and Clinical Practice*.

[20] Joh H., Oh J., Lee H., Kawachi I. (2013). Gender and socioeconomic status in relation to weight perception and weight control behavior in korean adults. *Obesity Facts*.

[21] Cleeman J. I. (2001). Executive summary of the third report of the National Cholesterol Education Program (NCEP) expert panel on detection, evaluation, and treatment of high blood cholesterol in adults (adult treatment panel III). *Journal of the American Medical Association*.

[22] Lee S. Y., Park H. S., Kim D. J. (2007). Appropriate waist circumference cutoff points for central obesity in Korean adults. *Diabetes Research and Clinical Practice*.

[23] Walker S. E., Gurka M. J., Oliver M. N., Johns D. W., DeBoer M. D. (2012). Racial/ethnic discrepancies in the metabolic syndrome begin in childhood and persist after adjustment for environmental factors. *Nutrition, Metabolism and Cardiovascular Diseases*.

[24] Gurka M. J., Lilly C. L., Oliver M. N., Deboer M. D. (2014). An examination of sex and racial/ethnic differences in the metabolic syndrome among adults: a confirmatory factor analysis and a resulting continuous severity score. *Metabolism: Clinical and Experimental*.

